# Bioelectrical impedance phase angle is associated with physical performance before but not after simulated multi‐stressor military operations

**DOI:** 10.14814/phy2.15649

**Published:** 2023-03-22

**Authors:** Alyssa N. Varanoske, Melissa N. Harris, Callie Hebert, Neil M. Johannsen, Steven B. Heymsfield, Frank L. Greenway, Arny A. Ferrando, Jennifer C. Rood, Stefan M. Pasiakos

**Affiliations:** ^1^ Military Performance Division, U.S. Army Research Institute of Environmental Medicine Natick Massachusetts USA; ^2^ Oak Ridge Institute for Science and Education Oak Ridge Tennessee USA; ^3^ Pennington Biomedical Research Center Louisiana State University Baton Rouge Louisiana USA; ^4^ Department of Geriatrics, Donald W. Reynolds Institute on Aging, Center for Translational Research in Aging & Longevity University of Arkansas for Medical Sciences Little Rock Arkansas USA

**Keywords:** impedance, reactance, resistance, strength, testosterone

## Abstract

Physical performance decrements observed during multi‐stressor military operations may be attributed, in part, to cellular membrane dysfunction, which is quantifiable using phase angle (PhA) derived from bioelectrical impedance analysis (BIA). Positive relationships between PhA and performance have been previously reported in cross‐sectional studies and following longitudinal exercise training programs, but whether changes in PhA are indicative of acute decrements in performance during military operations is unknown. Data from the Optimizing Performance for Soldiers II study, a clinical trial examining the effects of exogenous testosterone administration on body composition and performance during military stress, was used to evaluate changes in PhA and their associations with physical performance. Recreationally active, healthy males (*n* = 34; 26.6 ± 4.3 years; 77.9 ± 12.4 kg) were randomized to receive testosterone undecanoate or placebo before a 20‐day simulated military operation, which was followed by a 23‐day recovery period. PhA of the whole‐body (Whole) and legs (Legs) and physical performance were measured before (PRE) and after (POST) the simulated military operation as well as in recovery (REC). Independent of treatment, PhA_Whole_ and PhA_Legs_ decreased from PRE to POST (*p* < 0.001), and PhA_Legs_, but not PhA_Whole_, remained lower at REC than PRE. PhA_Whole_ at PRE and REC were associated with vertical jump height and Wingate peak power (*p* < 0.001–0.050), and PhA_Whole_ at PRE was also associated with 3‐RM deadlift mass (*p* = 0.006). However, PhA at POST and changes in PhA from PRE to POST were not correlated with any performance measure (*p* > 0.05). Additionally, PhA was not associated with aerobic performance at any timepoint. In conclusion, reduced PhA from PRE to POST provides indirect evidence of cellular membrane disruption. Associations between PhA and strength and power were only evident at PRE and REC, suggesting PhA may be a useful indicator of strength and power, but not aerobic capacity, in non‐stressed conditions, and not a reliable indicator of physical performance during severe physiological stress.

## Graphical abstract

Military operations induce decrements in bioelectrical impedance analysis phase angle, indicating cellular membrane disruption. Phase angle was associated with strength and power performance, but not aerobic capacity, before and in recovery from the operation. However, changes in phase angle measured immediately after the operation were not associated with changes in performance, indicating that phase angle may be related to strength and power in non‐stressed conditions, but not a reliable indicator of performance during severe physiological stress.

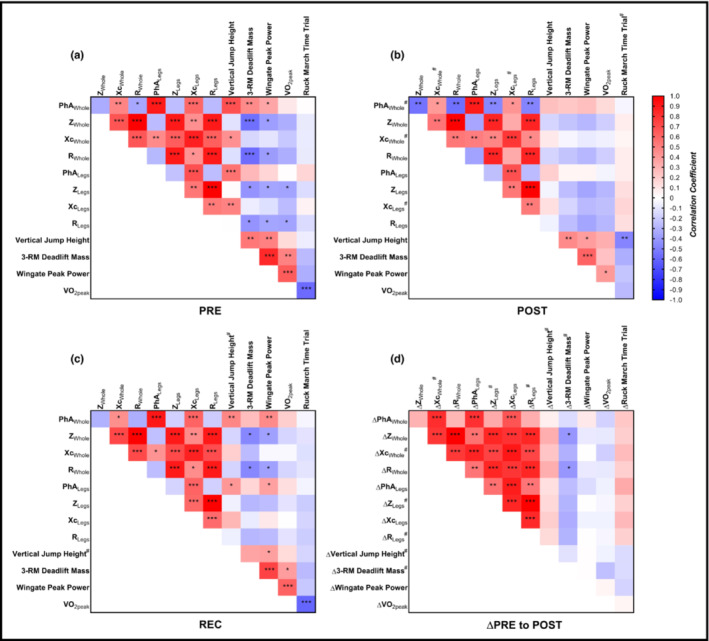

## 1 INTRODUCTION

Bioelectrical impedance analysis (BIA) is a non‐invasive, user‐friendly, inexpensive tool often used to estimate body composition changes in response to exercise training paradigms, dietary interventions, aging, and disease (Lukaski et al., 2017; Lukaski & Raymond‐Pope, 2021). BIA assesses the conductivity of the biological components of the human body by emitting a small electrical current into the body and measuring the passage of the current through the tissues (Kyle et al., 2004; Ward, 2019). The opposition to current flow, termed resistance (R), along with the current delay caused by cell membrane capacitance, termed reactance (Xc) (Kyle et al., 2004), are often used in population‐specific prediction equations to estimate fat‐free mass (FFM) and fat mass (Campa et al., 2021; Campa, Gobbo, et al., 2022; Lukaski & Raymond‐Pope, 2021). When expressed trigonometrically, the relationship between Xc and R is called impedance (Z), and the ratio of these variables presented in degrees is the phase angle (PhA). A change in bioelectrical impedance properties, including cellular membrane integrity or intracellular (ICW)/extracellular water (ECW) distribution directly affect Xc and R, resulting in a shift of the PhA (Francisco et al., 2020; Silva et al., 2014; Stobaus et al., 2012; Toso et al., 2000). Specifically, increases in body water result in decreased R (Campa, Colognesi, et al., 2022; Lukaski et al., 2019; Lukaski & Raymond‐Pope, 2021; Piccoli et al., 1994), whereas increases in body cell mass, cellular membrane integrity, and membrane storage capacity increase Xc (Stobaus et al., 2012). The interaction of Xc and R resulting in a high PhA therefore signifies greater body cell mass, cellular membrane integrity, and cellular function (Campa, Colognesi, et al., 2022; Custodio Martins et al., 2022).

PhA in the human body typically ranges from 1° to 12° (Sardinha & Rosa, 2023), and higher values within this range are positively associated with muscle strength and physical function (Cioffi et al., 2020; Custodio Martins et al., 2022; Fukuoka et al., 2022; Giorgi et al., 2018; Langer et al., 2022; Micheli et al., 2014; Norman et al., 2011; Piccoli et al., 2007; Rodriguez‐Rodriguez et al., 2016; Sato et al., 2020). Increases in PhA have been reported following resistance training programs and concurrent exercise training (Campa, Colognesi, et al., 2022; Sardinha & Rosa, 2023). Alternatively, low PhA has been reported in individuals presentingmalnutrition, alcoholism, cancer/cachexia, HIV, sarcopenia, and in older adults (Cardinal et al., 2010; Fukuoka et al., 2022; Genton et al., 2017, 2018; Gupta et al., 2008; Lukaski et al., 2017; Nescolarde et al., 2013; Tanaka et al., 2019; Toso et al., 2000). Taken together, low PhA is generally indicative of diminished cellular membrane integrity, physical fitness, and overall health, whereas high PhA indicates improved cellular function, strength, and quality of life (Custodio Martins et al., 2022; Lukaski et al., 2017; Norman et al., 2012). As such, PhA has been described as a global indicator of muscle cellular health and quality and is often used to predict functional health outcomes (Campa, Colognesi, et al., 2022; Lukaski et al., 2017; Lukaski & Raymond‐Pope, 2021).

Optimization of physical fitness, including muscular strength, power, and aerobic capacity, is an integral component of Warfighter training, as sustained, multi‐stressor military operations comprised of high energy expenditures, inadequate energy intake, and sleep deprivation typically degrade muscle mass and physical performance (Lieberman et al., 2002, 2005; Margolis et al., 2014; Nindl et al., 2002, 2007). It is generally well‐accepted that size is an essential determinant of the force‐generating capacity of muscle (Bamman et al., 2000); therefore, attenuating muscle mass loss during military operations may mitigate performance decline. However, the most accurate quantification of muscle mass often relies on invasive, expensive, technical, large, or radiation‐emitting equipment (i.e., dual‐energy x‐ray absorptiometry, magnetic resonance imaging, D‐3 creatinine dilution, air displacement plethysmography), making muscle mass quantification in the field impractical. Furthermore, even muscle mass estimations derived from less‐invasive, more convenient pieces of equipment, such as BIA, are based off population‐specific prediction equations which rely on inherent assumptions of cellular hydration that may not be appropriate for all individuals (Campa, Colognesi, et al., 2022). Examining changes in raw BIA parameters, such as PhA, R, Xc, and Z, may circumvent these issues by providing a non‐invasive method of elucidating cellular health, function, and membrane disruption to help predict performance during military operations without the use of population‐based prediction equations, costly equipment, or the use of radiation‐emitting devices for muscle mass estimation.

Despite the potential for PhA to elucidate the underlying causes of performance decline, previous research using this metric of muscle quality has been limited to examining relationships between PhA and physical performance in cross‐sectional studies (Fukuoka et al., 2022; Giorgi et al., 2018; Langer et al., 2022; Micheli et al., 2014; Norman et al., 2011; Piccoli et al., 2007; Rodriguez‐Rodriguez et al., 2016; Sato et al., 2020) or following longitudinal exercise training programs (Fukuda et al., 2016; Nunes et al., 2019; Ribeiro et al., 2017; Souza et al., 2017). Research in healthy, male Army cadets reports a positive relationship between PhA and handgrip strength (Langer et al., 2022); however, whether this relationship still exists following sustained military operations has not yet been explored. Furthermore, previous studies have demonstrated that the relationship between muscle mass and strength is not linear (Ahtiainen et al., 2016; Jakobsgaard et al., 2018; Mattocks et al., 2017; Parente et al., 2008), and decreases in muscle strength have been reported during simulated multi‐stressor military operations independent of muscle mass loss (Pasiakos et al., 2019; Varanoske et al., 2022). One such investigation highlighting the discordant relationship between muscle mass and strength was recently reported by our laboratory. Healthy, young males who were administered exogenous testosterone (750 mg testosterone undecanoate) prior to a 20‐day simulated military operation had greater FFM compared to controls at the end of the simulation; however, the preservation of FFM did not translate to physical performance enhancement (Varanoske et al., 2021, 2022). As military operations and training usually elicit physical performance decrements, muscle mass loss, inflammation, and muscle damage (Lieberman et al., 2002, 2005; Margolis et al., 2014; Nindl et al., 2002, 2007), it is possible that examining changes in raw BIA parameters may provide a non‐invasive method of elucidating cellular membrane disruption, which may undermine physical performance capabilities. Therefore, the purpose of this analysis was to examine the associations between changes in raw BIA parameters and physical performance following a simulated, sustained military operation. A secondary purpose was to determine the effects of exogenous testosterone administration (TA) on changes in raw BIA parameters following simulated, sustained military operations.

## 2 MATERIALS AND METHODS

This manuscript reports a secondary analysis of variables measured during a randomized, double‐blind, placebo‐controlled trial, which was designed to assess whether exogenous TA (750 mg undecanoate, administered once) restores eugonadal circulating total and free testosterone concentrations and improves measures of FFM and physical performance during, and in recovery from, a 20‐day simulated sustained military operation (Varanoske et al., 2021, 2022). The study design and methodology relating to primary outcomes have been previously described (Varanoske et al., 2021, 2022) but are summarized below to provide context for the outcomes discussed in this report. All testing occurred at the Pennington Biomedical Research Center (PBRC) in Baton Rouge, LA. The Institutional Review Board (IRB) of the PBRC (protocol 2019‐017) and the U.S. Army Medical Research and Development Command, Human Research Protections Office approved the study protocol and trial documents. All procedures were in accordance with the ethical standards of the 1964 Helsinki Declaration and its later amendments. The study occurred from October 2019 through July 2021. The ClinicalTrials.gov identifier is NCT04120363.

### 2.1 Participants

Complete inclusion and exclusion criteria and extended details regarding participant recruitment, randomization, and attrition have been reported previously (Varanoske et al., 2021, 2022). Briefly, males aged 18–35 who were healthy, physically active (expended at least 300 kcal/day on average through structured aerobic and strength‐training activities), had normal testosterone concentrations (10.4–34.7 nmol/L), and met age‐specific U.S. Army body composition standards (Department of the Army Headquarters, 2013) were recruited.

### 2.2 Experimental design

Participants underwent a 3‐phase, 50‐day study, consisting of 7 days of baseline testing (days 1–7), 20 days of simulated military operations (days 8–27), and 23 days of recovery (days 28–50) (details in Varanoske et al., 2021, 2022). After completing baseline testing (day 8), participants were randomized to receive either a single intramuscular injection of testosterone undecanoate (TEST; AVEED™, 750 mg testosterone undecanoate in 3 mL) or an iso‐volumetric placebo (PLA; sesame oil solution, 3 mL). Details of randomization, treatment allocation, and blinding have been published (Varanoske et al., 2021). The baseline and recovery periods consisted of controlled feeding and daily check‐ins, but participants were permitted to self‐select their physical activity and sleep patterns. The 20‐day sustained military operation was a highly‐controlled diet, physical activity, and sleep intervention (participants lived in the inpatient unit at PBRC), which consisted of four consecutive 5‐day cycles of undulating stress. The first 2 days in each cycle were ‘low stress' days, entailing exercise‐induced energy expenditures equaling 1000 kcal above baseline values and 8 h of sleep per night. The following 3 days in each cycle were ‘high stress' days, entailing exercise‐induced energy expenditures equaling 3000 kcal above baseline values and 4 h of sleep per night. To reach this energy expenditure during the simulated military operation, participants exercised several times per day using a variety of endurance and muscle‐loading modalities to mimic movements typically observed during real‐life sustained military operations. Steady‐state ruck marching was the primary exercise modality, but other activities included walking, running, cycling, elliptical, field‐based operational activities, and stretching (see (Varanoske et al., 2021) for details). Daily energy expenditure was determined for each participant and exercise using the Compendium of Physical Activities (Ainsworth et al., 2011) or according to published equations (American College of Sports Medicine et al., 2018; Glass et al., 2007).

Throughout all phases, total energy intake and macronutrient distribution remained fixed but were individualized for each participant. Individual physical activity patterns and habitual exercise‐induced energy expenditure during and prior to the baseline period were determined using accelerometry and a physical activity questionnaire (PAR‐Q+). Resting metabolic rate was measured by using indirect calorimetry (Deltatrac II Metabolic Cart Sensormedics, Yorba Linda, CA). The food records and resting metabolic rate measurements were used to calculate total daily energy expenditure and prescribe individual dietary intake to maintain energy balance and body mass. The macronutrient distribution of the diet was based on the composition of the Meal, Ready‐to‐Eat (MRE) (15% protein, 55% carbohydrate, and 30% fat) (Varanoske et al., 2021), the standard US Department of Defense ration. Dietary intake during the simulated military operation was monitored on the in‐patient unit but consisted solely of items derived from MREs (menu 39; Ameriqual, Evansville, IN, USA). For the baseline and recovery phases, Registered Dietitians developed individualized menus consisting of commercial products, and compliance was checked daily.

#### 2.2.1 Outcome measures

Procedural details of all outcome variables have been previously reported (Varanoske et al., 2021), but those pertaining to this analysis are summarized below.

### 2.3 Bioelectrical impedance analysis (BIA) procedures

Participants arrived at the body composition testing facility on the morning of days 7 (PRE), 28 (POST), and 49 (REC) after at least an 8 hour (overnight) fast. As the data presented here was a secondary analysis of a larger investigation, day 49 was selected as the REC timepoint to allow for sufficient rest between physical performance testing (days 46–47) and body composition testing and to ensure that body composition testing was conducted prior to muscle and whole‐body protein turnover analyses (days 49–50) (Varanoske et al., 2021). Participants were encouraged to hydrate properly on the day leading up to and the morning of the assessment. Hydration status was evaluated upon arrival via analysis of urine specific gravity (CLINITEK 500, Siemens Healthcare Diagnostics, Malvern, PA, USA) prior to BIA analyses. Upon confirmation, participants were instructed to remove footwear, socks, and jewelry and were placed supine on an examination table for at least 5 minutes prior to examination. Contact sites for electrodes on the fingers and ankles were cleaned before measurement with a sterile antimicrobial tissue provided by the manufacturer. Eight touch type electrodes were used in accordance with standard protocols. Whole‐body segmented multi‐frequency BIA measurements were acquired on an InBody S10 system (InBody Inc., Cerritos, CA). Segmental (right leg, left leg, right arm, left arm, trunk) impedance (Z), reactance (Xc), and phase angle (PhA) values at 50 khz were recorded directly from the device. Segmental resistance (R) values were calculated from segmental Z and Xc according to the following equation:

$$Z=\surd \left(R^{2}+{\mathrm{Xc}}^{2}\right).$$

Whole‐body Z, Xc, and R (Z_Whole_, Xc_Whole_, R_Whole_, respectively) were calculated from the sum of segmental right leg, right arm, and trunk values according to manufacturer's instructions. Whole‐body PhA (PhA_Whole_) was calculated using the following equation:

$$\begin{array}{c} {\mathrm{PhA}}_{\text{Whole}}=\arctan\left(\frac{{\mathrm{Xc}}_{\text{Whole}}}{R_{\text{Whole}}}\right)\times \left(\frac{180}{\pi }\right) \end{array}.$$

As recent research suggests that PhA of the lower body is a better predictor of physical performance than PhA_Whole_ (Bongiovanni et al., 2021), Z, Xc, R, and PhA of the legs (Z_Legs_, Xc_Legs_, R_Legs_, and PhA_Legs_, respectively) were also calculated using the average of the left and right legs for each parameter.

### 2.4 Physical performance

A battery of physical performance tests was completed during each phase, as previously described (Varanoske et al., 2021). Participants were familiarized with each test before testing in each phase. The order and timing of the tests were standardized, and participants completed a dynamic warm‐up before testing began. For the analysis in this report, one variable was chosen from each of the five tests based on what was most encompassing and commonly reported in the literature. A brief summary of each test and the measures chosen are reported below.

The vertical jump test was used to evaluate lower‐body power (Vertec, Jump USA, Sunnyvale, CA, USA). Participants completed a series of three maximal countermovement jumps by flexing their knees and hips, moving downward, and extending their knees and hips rapidly while swinging up their dominant arm to touch the highest vane on the Vertec. Jump height from the jump in which participants reached the greatest height was used in the analysis.

A three repetition maximum (3‐RM) trap bar deadlift was used to assess total body muscular strength in accordance with the U.S. Army Combat Fitness Test (Department of the Army Headquarters, 2017). Following three warm‐up sets, the bar was loaded with ~85% of their estimated 1‐RM. Participants stood in the middle of the bar with feet shoulder width apart, bent at the knees and hips, reached down, and grasped the center of the handles. They then stood up and lifted the bar by extending the hips and knees until in an upright stance, paused slightly at the top of the movement, flexed the hips and knees slowly, and lowered the bar to the ground in a controlled manner. If they failed to complete three repetitions, they retested at a lower weight. If successful, additional weight was added, and they retested after 3 min of rest. The maximal amount of mass they could lift during the 3‐RM deadlift was recorded.

The Wingate test was used to measure anaerobic capacity. Participants were positioned on an electronically braked cycle ergometer (Excalibur Sport, Lode, The Netherlands) equipped with software (Lode Ergometry Manager software version 10.11.0, Lode B.V., Lode, The Netherlands) and began pedaling for 5 min at 50 W. On “Go,” participants increased their cadence to 90 rpm, and a fixed resistance based off body mass, cycle cadence, and torque factor was added to the bike. Participants were instructed to pedal maximally for the duration of the 30s test. Absolute peak power was recorded, as the reported decrease in body mass from PRE to POST artificially influenced relative peak power measures.

Peak aerobic capacity (i.e., VO_2peak_) was measured using a graded exercise test and an indirect open circuit respiratory system (ParvoMedics TrueOne 2400, East Sandy, UT, USA) on a treadmill (Track Master TMX425CP, Full Vision, Inc., Newton, KS, USA). Participants began by completing a 5‐min warm‐up and then ran for 4 min at a pace predetermined during familiarization at a 0% grade. The grade was then increased to 2%, followed by an additional 2% every 2 min thereafter until volitional exhaustion. Absolute VO_2peak_ was recorded for the same aforementioned reason of avoiding the use of relative measures.

A 4‐km (2.5 mile) outdoor timed ruck march (e.g., backpack load carriage) was completed while wearing a 31.3 kg rucksack to assess military‐relevant aerobic endurance. Total time to complete the march was recorded.

### 2.5 Statistical analysis

Sample size was based off the primary outcome, anticipated differences in lower‐body physical performance in TEST relative to PLA, as previously described (Varanoske et al., 2021, 2022). As the present analysis represent secondary outcomes for the larger study, no a priori power analysis was performed. Changes in Xc, R, Z, PhA, and physical performance over time were assessed using a mixed‐effect linear model analysis of variance (ANOVA). Subject was treated as a random effect, and group (TEST and PLA), time (PRE, POST, and REC), and group × time interaction were considered fixed effects in the model. Least squares means from the model were used to estimate interaction effects. Distribution and heterogeneity of residuals were examined, and non‐normal data were log_10_‐transformed as needed to meet model assumptions. When a significant main effect or interaction was detected, pairwise comparisons across time points were conducted, and *p*‐values were adjusted using the Bonferroni correction. Outliers were identified (values exceeding 2 × interquartile range above the 75th percentile or below the 25th percentile) for the primary outcome variable (PhA) and were excluded from analyses (*n* = 1 at POST). As group × time interactions were not significant for any variable, treatment groups were pooled for correlation analyses at all time points. Mean differences between raw BIA vectors in Whole and Legs at each time point were compared using paired samples *t*‐tests. Associations between BIA parameters and physical performance at PRE, POST, and REC, as well as changes in these variables from PRE to POST (i.e., POST value – PRE value) were assessed using Pearson's correlations.

All analyses were considered 2‐tailed, with *α* = 0.05 considered statistically significant and were completed with statistical software (SPSS V.26.0, Chicago, IL, USA). Unless otherwise noted, data following a normal distribution are reported as estimated mean ± estimated standard error, and data that were log_10_‐transformed are reported as geometric estimated mean ± geometric estimated standard error.

## 3 RESULTS

### 3.1 Participants

Physically active males were enrolled (*n* = 34), randomized (*n* = 34; TEST: *n* = 16; PLA: *n* = 18), and completed the study (*n* = 32; TEST: *n* = 16; PLA: *n* = 16; participant flow chart presented previously in Varanoske et al., 2022). No group demographic differences were observed at PRE (all *p* > 0.05; Table 1; full details previously reported in Varanoske et al., 2022). Two participants in the PLA group dropped out of the study during the simulated military operations and were therefore not included in the analyses except for correlations between variables at PRE.

**TABLE 1:** Participant demographic data at the start of PRE.

	TEST (*n* = 16)	PLA (*n* = 18)	Total (*n* = 34)	*p*‐value
Age (years)	27.1 ± 4.3	26.2 ± 4.5	26.6 ± 4.3	0.646
Body mass (kg)	77.7 ± 14.5	78.1 ± 10.6	77.9 ± 12.4	0.924
Height (cm)	177.1 ± 8.1	176.3 ± 5.3	176.7 ± 6.7	0.761

*Note*: Data were analyzed using independent samples *t*‐tests, and values are presented as mean ± standard deviation.

Abbreviations: PLA, participants randomized to 750 mg sesame oil solution on day 8; PRE, before the 20‐day simulated, multi‐stressor military operation; TEST, participants randomized to 750 mg testosterone undecanoate on day 8.

### 3.2 Longitudinal responses to the simulated military operation intervention and recovery

**FIGURE 1:**
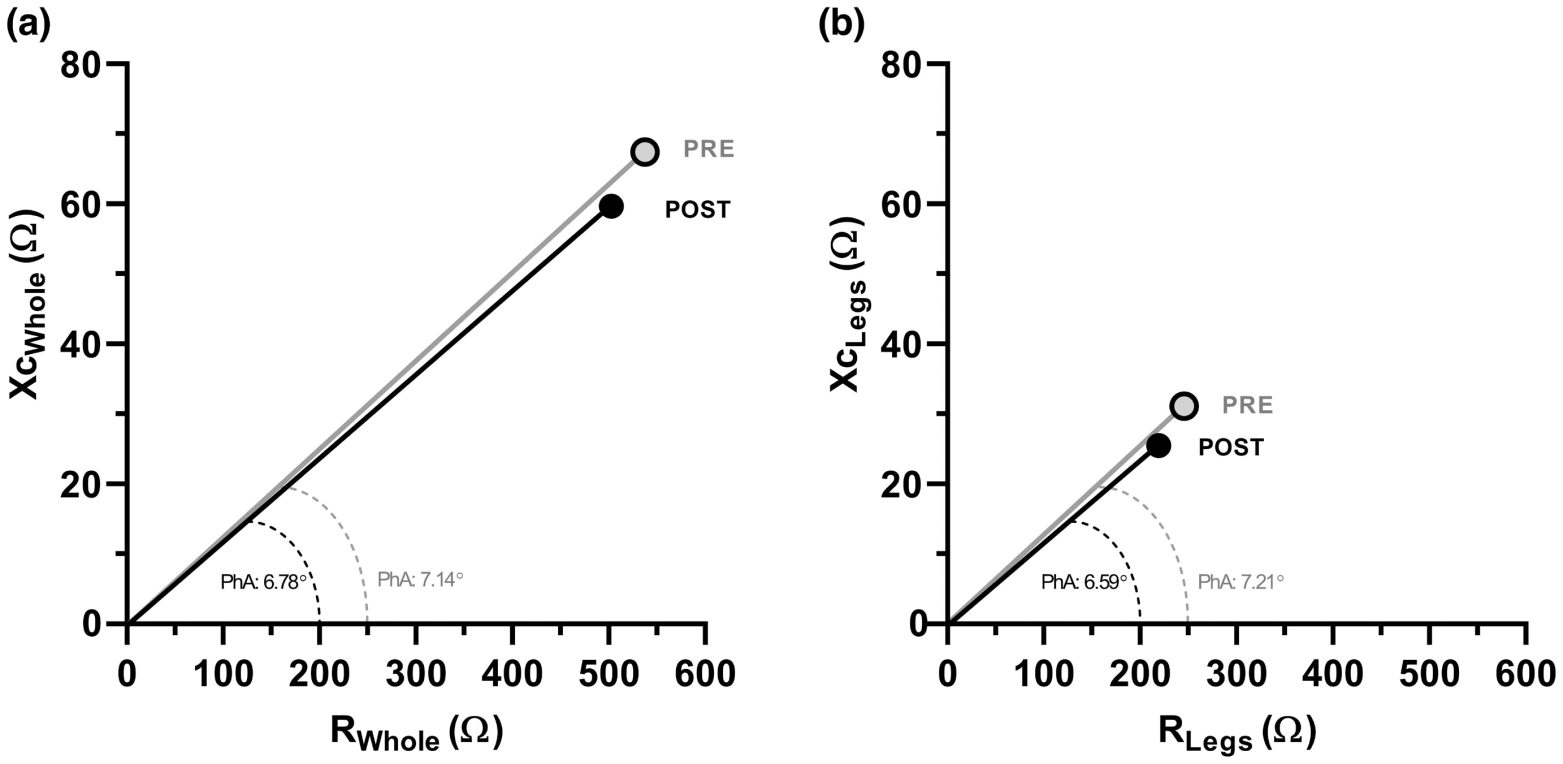
Depiction of the bioelectrical analysis vector displacements from PRE to POST in (a) the whole body and (b) average of the left and right legs. Data are presented as the means of TEST and PLA. Note that angles are not to scale. PhA, phase angle; PLA, participants randomized to 750 mg sesame oil solution on day 8; POST, after the 20‐day simulated, multi‐stressor military operation; PRE, before the 20‐day simulated, multi‐stressor military operation; R, resistance; TEST, participants randomized to 750 mg testosterone undecanoate on day 8; Xc, reactance.

**FIGURE 2:**
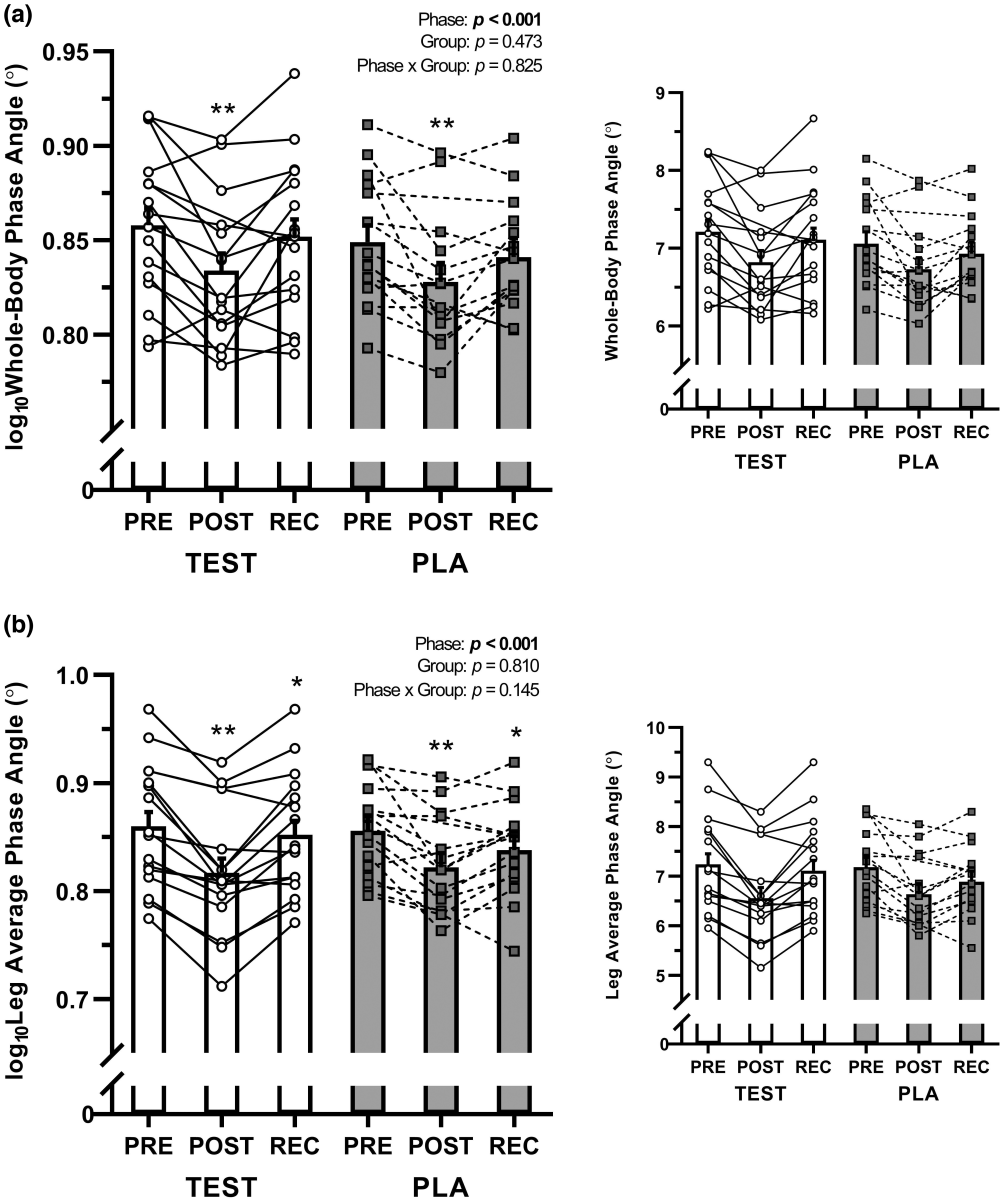
Changes in (a) whole‐body phase angle and (b) the average of the left and right leg phase angle during each study phase. Data were analyzed using mixed‐model analyses of variances (ANOVA) with subject as a random effect, and group (TEST and PLA), time (PRE, POST, and REC), and group × time interaction were considered fixed effects in the model. As both the whole‐body and average leg phase angle data were non‐normally distributed, data were log_10_‐transformed for analysis. Values are presented as the transformed data on the left and the raw data on the right for interpretation. Circles and squares represent individual data points, and bar graphs are presented as geometric estimated mean ± standard error. PLA, participants randomized to 750 mg sesame oil solution on day 8; TEST, participants randomized to 750 mg testosterone undecanoate on day 8. Main effect of time: *Different from PRE and POST (*p* < 0.05); **Different from PRE and REC (*p* < 0.001).

A main effect of phase was observed for all BIA vectors examined (all *p* < 0.001) (Table 2, Figure 1). Raw impedance vectors for the whole‐body and legs (Z_Whole_, Z_Legs_, Xc_Whole_, Xc_Legs_, R_Whole_, R_Legs_) decreased from PRE to POST (all *p* < 0.001) and increased from POST to REC (*p* < 0.001 to 0.027) but remained lower than PRE values by the end of the study (all PRE vs. REC *p* < 0.005). PhA_Whole_ and PhA_Legs_ were decreased from PRE to POST (both *p* < 0.001) and increased from POST to REC (both *p* < 0.001). PhA_Whole_ was not different from PRE at REC (*p* = 0.194), but PhA_Legs_ remained lower at REC compared to PRE (*p* = 0.022) (Figure 2). No group differences between groups or phase × group interactions were observed for any BIA parameter (all *p* > 0.05).

**TABLE 2:** Changes in BIA parameters for each study phase.

	Group	Phase			*P*‐value		
		PRE	POST	REC	Phase	Group	Phase × group
PhA_Whole_^#^ (°)	TEST	7.21 ± 0.15	6.82 ± 0.15	7.11 ± 0.15	**<0.001** ^**1,2**^	0.473	0.825
	PLA	7.06 ± 0.15	6.73 ± 0.15	6.93 ± 0.15			
	Total	7.14 ± 0.10	6.78 ± 0.11	7.01 ± 0.11			
Z_Whole_ (Ω)	TEST	542.9 ± 14.3	504.3 ± 14.4	529.7 ± 14.3	**<0.001** ^**3**^	0.825	0.244
	PLA	540.9 ± 14.3	508.4 ± 14.5	514.7 ± 14.4			
	Total	541.9 ± 10.1	506.3 ± 10.2	522.2 ± 10.2			
Xc_Whole_ (Ω)	TEST	68.1 ± 1.8	59.9 ± 1.8	65.6 ± 1.8	**<0.001** ^**3**^	0.443	0.303
	PLA	66.7 ± 1.8	59.6 ± 1.8	62.2 ± 1.8			
	Total	67.4 ± 1.3	59.7 ± 1.3	63.9 ± 1.3			
R_Whole_ (Ω)	TEST	538.5 ± 14.3	500.7 ± 14.4	525.6 ± 14.3	**<0.001** ^**3**^	0.833	0.246
	PLA	536.8 ± 14.3	504.8 ± 14.5	510.9 ± 14.4			
	Total	537.7 ± 10.1	502.8 ± 10.2	518.3 ± 10.1			
PhA_Legs_^#^ (°)	TEST	7.24 ± 0.21	6.56 ± 0.21	7.11 ± 0.21	**<0.001** ^**3**^	0.810	0.145
	PLA	7.18 ± 0.21	6.64 ± 0.21	6.89 ± 0.21			
	Total	7.21 ± 0.15	6.59 ± 0.15	7.00 ± 0.15			
Z_Legs_ (Ω)	TEST	246.1 ± 6.4	216.0 ± 6.4	235.4 ± 6.4	**<0.001** ^**3**^	0.715	0.167
	PLA	249.3 ± 6.4	225.5 ± 6.5	231.9 ± 6.4			
	Total	247.7 ± 4.5	220.7 ± 4.6	233.6 ± 4.5			
Xc_Legs_ (Ω)	TEST	31.1 ± 1.0	24.8 ± 1.0	29.3 ± 1.0	**<0.001** ^**3**^	0.942	0.084
	PLA	31.3 ± 1.0	26.2 ± 1.0	27.9 ± 1.0			
	Total	31.2 ± 0.7	25.5 ± 0.7	28.6 ± 0.7			
R_Legs_ (Ω)	TEST	244.1 ± 6.3	214.6 ± 6.4	233.6 ± 6.3	**<0.001** ^**3**^	0.712	0.171
	PLA	247.3 ± 6.3	223.9 ± 6.5	230.2 ± 6.4			
	Total	245.7 ± 4.5	219.2 ± 4.6	231.9 ± 4.5			

*Note*: Raw and log_10_‐transformed data were analyzed using mixed‐model analyses of variances (ANOVA) with subject as a random effect, and group (TEST and PLA), time (PRE, POST, and REC), and group × time interaction were considered fixed effects in the model. Values are estimated mean ± standard error for normally‐distributed data or geometric estimated mean ± standard error for log_10_‐transformed data (^#^Data is log_10_‐transformed for analysis). *p*‐values in bold are significant (*p* < 0.05): ^1^PRE is different from POST, ^2^POST is different from REC, ^3^All phases are different.

Abbreviations: BIA, bioelectrical impedance analysis; Legs, average of left and right legs; PhA, phase angle; PLA, participants randomized to 750 mg sesame oil solution on day 8; POST, after the 20‐day simulated, multi‐stressor military operation; PRE, before the 20‐day simulated, multi‐stressor military operation; R, resistance; REC, after the 23‐day recovery period; TEST, participants randomized to 750 mg testosterone undecanoate on day 8; Whole, whole‐body; Xc, reactance; Z, impedance.

Changes in physical performance variables throughout this study have previously been reported in the intent‐to‐treat population (a larger sample of participants) (see Figure 5 and Supplementary Tables 2–6 in Varanoske et al., 2022). For context in this report, these metrics have been re‐analyzed in the smaller sample of participants. A main effect of phase was observed for all physical performance parameters (*p* < 0.001 to 0.017) (Table 3). Vertical jump height, 3‐RM deadlift mass, Wingate peak power decreased, and ruck march time trial time increased, from PRE to POST (vertical jump height: *p* < 0.001; 3‐RM deadlift mass: *p* = 0.027, Wingate peak power: *p* < 0.001; ruck march time trial: *p* < 0.001). Wingate peak power increased (*p* = 0.010), and ruck march time trial decreased (*p* < 0.001) from POST to REC and were not different from PRE at REC (Wingate peak power: *p* = 0.082; ruck march time trial: *p* = 0.822). Vertical jump height and 3‐RM deadlift mass were not different at REC from POST (vertical jump height: *p* = 0.089; 3‐RM deadlift mass: *p* = 0.066) or PRE (vertical jump height: *p* = 0.185; 3‐RM deadlift mass: *p* > 0.999). VO_2peak_ was increased from POST to REC (*p* < 0.001), but was not different between PRE and POST (*p* = 0.204) or PRE and REC (*p* = 0.058).

**TABLE 3:** Changes in physical performance parameters for each study phase.

	Group	Phase			*p*‐value		
		PRE	POST	REC	Phase	Group	Phase × group
Vertical jump height (cm)	TEST	53.6 ± 2.4	48.4 ± 2.4	52.8 ± 2.5	**<0.001** ^**1**^	0.970	0.258
	PLA	54.6 ± 2.4	49.9 ± 2.5	50.7 ± 2.4			
	Total	54.1 ± 1.7	49.1 ± 1.7	51.8 ± 1.7			
3‐RM deadlift mass (kg)	TEST	127.4 ± 7.0	122.7 ± 7.0	125.7 ± 7.2	**0.017** ^**1**^	0.615	0.344
	PLA	133.8 ± 7.0	122.4 ± 7.1	133.9 ± 7.0			
	Total	130.6 ± 5.0	122.5 ± 5.0	129.8 ± 5.0			
Wingate peak power, absolute^#^ (W)	TEST	843.3 ± 48.3	731.1 ± 48.3	792.5 ± 48.3	**<0.001** ^**1,2**^	0.789	0.985
	PLA	822.2 ± 48.3	717.8 ± 48.7	778.0 ± 48.3			
	Total	833.7 ± 34.2	724.4 ± 34.3	785.2 ± 34.2			
VO_2peak_, absolute (L/min)	TEST	3.21 ± 0.15	3.13 ± 0.15	3.29 ± 0.15	**<0.001** ^**2**^	0.621	0.664
	PLA	3.29 ± 0.15	3.21 ± 0.16	3.45 ± 0.15			
	Total	3.26 ± 0.11	3.17 ± 0.11	3.37 ± 0.11			
Ruck march time trial^#^ (min)	TEST	44.8 ± 2.1	50.0 ± 2.1	44.1 ± 2.1	**<0.001** ^**1,2**^	0.066	0.680
	PLA	40.5 ± 2.1	45.9 ± 2.1	38.5 ± 2.1			
	Total	42.6 ± 1.5	47.9 ± 1.5	41.2 ± 1.5			

*Note*: Physical performance data reanalyzed from Varanoske et al. (2022). Raw and log_10_‐transformed data were analyzed using mixed‐model analyses of variances (ANOVA) with subject as a random effect, and group (TEST and PLA), time (PRE, POST, and REC), and group × time interaction were considered fixed effects in the model. Values are estimated mean ± standard error for normally‐distributed data or geometric estimated mean ± standard error for log_10_‐transformed data (^#^Data is log_10_‐transformed for analysis). *p*‐values in bold are significant (*p* < 0.05): ^1^PRE is different from POST, ^2^POST is different from REC.

Abbreviations: PLA, participants randomized to 750 mg sesame oil solution on day 8; POST, after the 20‐day simulated, multi‐stressor military operation; PRE, before the 20‐day simulated, multi‐stressor military operation; REC, after the 23‐day recovery period; TEST, participants randomized to 750 mg testosterone undecanoate on day 8.

When groups were pooled, PhA_Whole_ was significantly different from PhA_Legs_ at POST (*p* = 0.036), but was not different at PRE (*p* = 0.117) and REC (*p =* 0.951). All other raw BIA parameters were significantly lower in Legs than Whole at all timepoints (*p* < 0.001).

### 3.3 Associations between BIA parameters and physical performance outcomes

#### 3.3.1 PRE, POST, and REC

**FIGURE 3:**
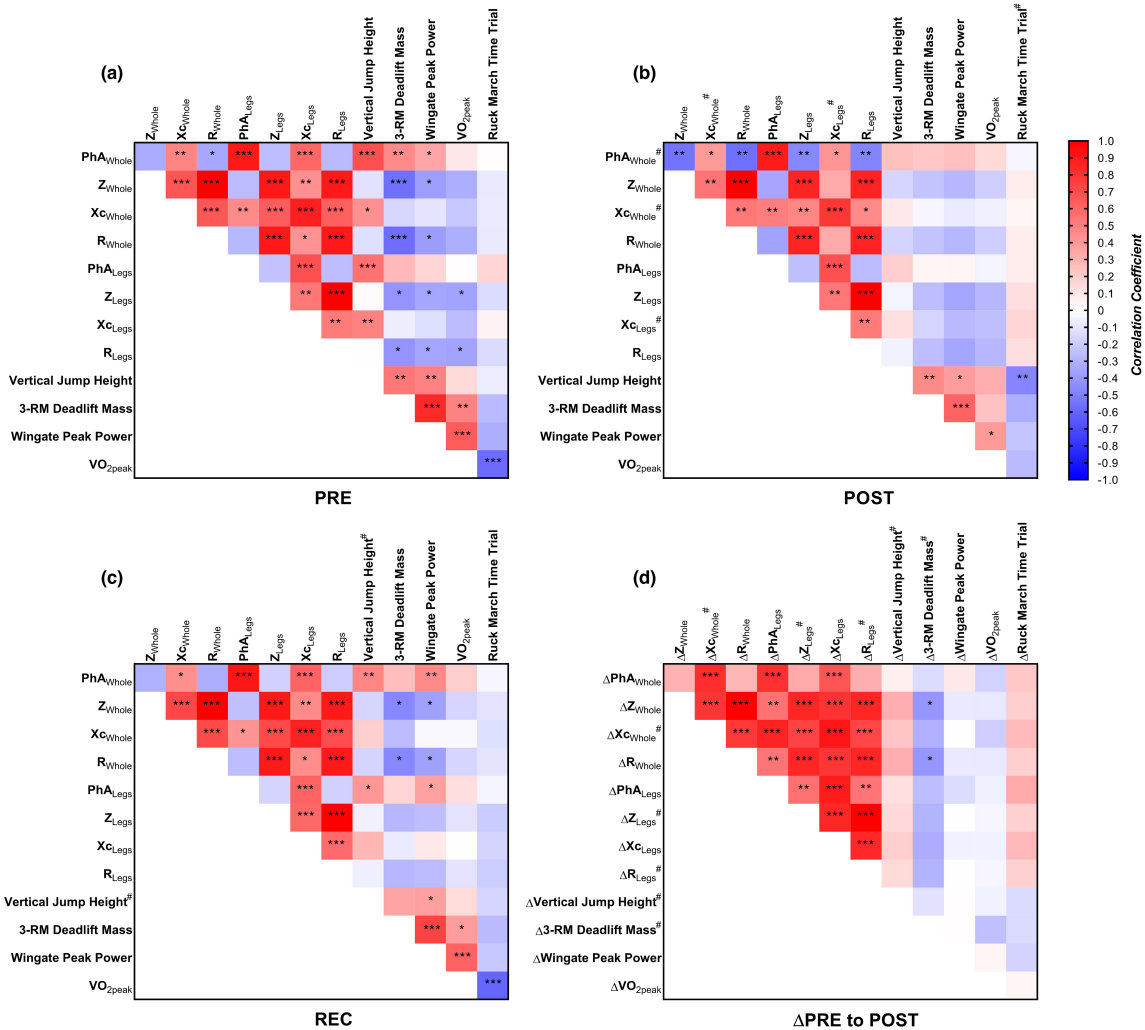
Heat map of associations between dependent variables at (a) PRE, (b) POST, (c) REC, and (d) changes from PRE to POST. Cells are colored based on the Pearson's correlation coefficient between the two variables. ^#^Data is log_10_‐transformed for analysis. *p < 0.05, **p < 0.01, ***p < 0.001. Legs, average of left and right legs; PhA, phase angle; POST, after the 20‐day simulated, multi‐stressor military operation; PRE, before the 20‐day simulated, multi‐stressor military operation; R, resistance; REC, after the 23‐day recovery period; Whole, whole‐body; Xc, reactance; Z, impedance.

**FIGURE 4:**
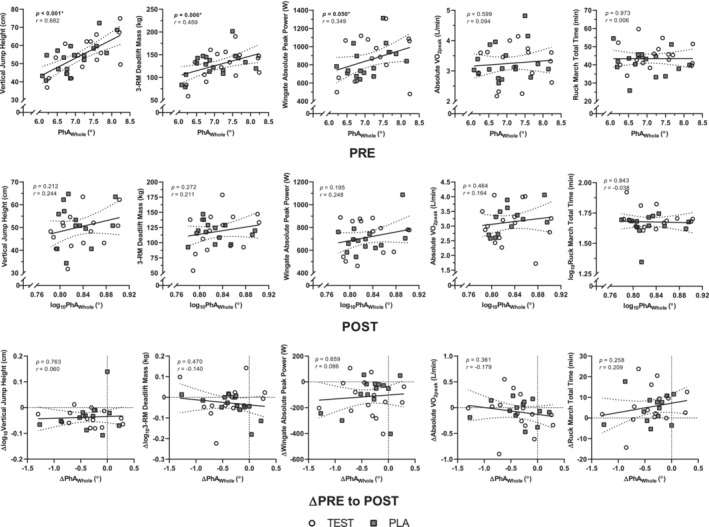
Correlations between PhA_Whole_ and physical performance variables at PRE, POST, and changes from PRE to POST. Circles and squares represent individual data points, solid lines represent the line of best fit, and dotted lines represent 95% confidence intervals of the line of best fit. PhA, phase angle; PLA, participants randomized to 750 mg sesame oil solution on day 8; POST, after the 20‐day simulated, multi‐stressor military operation; PRE, before the 20‐day simulated, multi‐stressor military operation; TEST, participants randomized to 750 mg testosterone undecanoate on day 8.

A heat map of the relationships between BIA parameters and physical performance outcomes are presented in Figures 3a–c, and correlation plots between PhA_Whole_ and physical performance variables at PRE and POST are presented in Figure 4. In all phases, Whole raw BIA parameters were significantly associated with their respective parameters in Legs (*r* = 0.784 to 0.911; all *p* < 0.001). Z_Whole_, R_Whole_, and Xc_Whole_ as well as Z_Legs_, R_Legs_, and Xc_Legs_ were positively correlated during all phases (*r* = 0.518 to >0.999, *p* < 0.001 to 0.004). PhA_Whole_ was positively correlated with Xc_Whole_ (*r* = 0.398 to 0.482; *p* = 0.004 to 0.033), and PhA_Legs_ was positively correlated with Xc_Legs_ in all phases (*r* = 0.684 to 0.690; all *p* < 0.001). PhA_Whole_ was negatively correlated with Z_Whole_ (*r* = −0.552, *p* = 0.002) and R_Whole_ (*r* = −0.561, *p* = 0.002) in POST, R_Whole_ in PRE (*r* = −0.344, *p* = 0.046), and neither in REC. PhA_Legs_ was not associated with Z_Legs_ or R_Legs_ in any phase.

PPhA_Whole_ and PhA_Legs_ were positively associated with vertical jump height (*r* = 0.408 to 0.682, *p* < 0.001 to 0.025) at PRE and REC as well as Wingate peak power at REC (*r* = 0.361 to 0.451, *p* = 0.011 to 0.046). PhA_Whole_ was also correlated with 3‐RM deadlift mass (*r* = 0.459, *p* = 0.006) and Wingate peak power (*r* = 0.349, *p* = 0.050) at PRE. R_Whole_, R_Legs_, Z_Whole_, and Z_Legs_ were negatively correlated with 3‐RM deadlift mass and Wingate peak power (*r* = −0.571 to −0.359, *p* < 0.001 to 0.044), and R_Legs_ and Z_Legs_ were also negatively correlated with VO_2peak_ (*r* = −0.366 to −0.364, *p* = 0.033 to 0.034) at PRE. Additionally, Xc_Whole_ and Xc_Legs_ were positively correlated with vertical jump height (*r* = 0.432, *p* = 0.011; *r* = 0.488, *p* = 0.003, respectively) at PRE. Z_Whole_ and R_Whole_, but not Z_Legs_ or R_Legs_, were negatively correlated with 3‐RM deadlift mass and Wingate peak power (*r* = −0.468, *p* = 0.012 and *r* = −0.370, *p* = 0.040, respectively) at REC. No raw BIA vectors were associated with any physical performance variable (all *p* > 0.05) at POST.

#### 3.3.2 Changes in variables throughout the simulated military operation

A heat map of the relationships between changes in BIA parameters and physical performance outcomes from PRE to POST is presented in Figure 3d, and correlation plots between changes in PhA_Whole_ and physical performance variables from PRE to POST are presented in Figure 4. Changes in all Whole raw BIA parameters were significantly associated with their respective parameters in Legs (*r* = 0.809 to 0.911; all *p* < 0.001). ΔZ, ΔR, and ΔXc in Whole and Legs were all positively correlated (*r* = 0.542 to >0.999, *p* < 0.001 to 0.002). ΔPhA_Whole_ and ΔPhA_Legs_ were positively correlated with ΔXc (*r* = 0.823 and 0.890, respectively; both *p* < 0.001). ΔPhA_Legs_ was also positively correlated with ΔZ_Legs_ and ΔR_Legs_ (*r* = 0.556, *p* = 0.002 and *r* = 0.544, *p* = 0.002, respectively).

ΔZ_Whole_ and ΔR_Whole_ were negatively correlated with Δ3‐RM deadlift mass (both *r* = −0.412, *p* = 0.026). Changes in all other variables, including ΔPhA_Whole_ and ΔPhA_Legs_, were not associated with changes in any physical performance variable (all *p* > 0.05).

## 4 DISCUSSION

The current analysis used a 20‐day, simulated sustained military operation as a model for studying changes in raw BIA metrics and the utility of correlating these measures with physical performance during, and in recovery from severe physiological stress in healthy, young males. Additionally, the potential protective effects of administering a single dose of testosterone undecanoate (750 mg) prior to the simulated sustained military operation on non‐invasive measures of muscle cellular integrity were examined. We report significant decreases in all raw BIA parameters measured (R, Xc, Z, and PhA) in both the whole body and legs from PRE to POST, which were not different between TEST and PLA. With the exception of PhA_Whole_, all of these parameters remained suppressed from PRE values at REC, 23 days after the end of the sustained military operation. Additionally, PhA_Whole_ was positively associated with performance on the vertical jump, 3‐RM deadlift, and Wingate tests, but not on the VO_2peak_ or ruck march time trial time tests at PRE. These associations persisted at REC with the exception the 3‐RM deadlift task. However, PhA was not associated with performance on any test at POST, and decreases in PhA from PRE to POST were not associated with decreases in physical performance. Taken together, these observations suggest that simulated sustained military operations disrupt cellular membrane integrity, and these consequences are not mitigated by TA. Also, PhA is significantly associated with strength and power performance, but not aerobic capacity in healthy, young males, and not during periods of acute stress or muscle damage.

To our knowledge, this is the first study to report changes in raw BIA parameters in response to military operations or training. It is also the first study to examine longitudinal BIA changes in response to TA in healthy, young males. Importantly, R, Xc, Z, and PhA are variables measured directly by the BIA device, and, unlike other BIA‐derived variables that require prediction equations to estimate/quantify [e.g., FFM, fat mass, percent body fat, total body water (TBW), ECW, ICW], they do not rely on inherent assumptions that may be inappropriate for certain individuals, increasing the chance of measurement error (Campa, Colognesi, et al., 2022). The decreases in R, Xc, Z, and PhA from PRE to POST are consistent with the physiologic effects of sustained military operations (Margolis et al., 2014; McClung et al., 2013; Nindl et al., 2002, 2007) and suggest changes in body composition, hydration status, cellular integrity, inflammatory status, muscular health, and injury (Lukaski et al., 2017; Nescolarde et al., 2013; Norman et al., 2012; Stobaus et al., 2012; Toso et al., 2000). Though the magnitude of cellular disruption, muscle damage, and inflammation induced by military operations is influenced by several factors including the extent of energy deficit, the duration of the operation, participant training status, sleep restriction, psychological distress, environmental conditions, gender, and physiological preparedness, our findings align with other studies using more invasive techniques such as muscle biopsies and blood draws to characterize the physiological impacts of military training on cellular health and muscle damage (Henning et al., 2011; Howard et al., 2020; Koury et al., 2016; Margolis et al., 2014; McClung et al., 2013; Pasiakos et al., 2019; Varanoske et al., 2018). Reductions in R generally indicate greater body fluid retention, which can be caused by either positive (increased lean body mass, euhydration) or negative (edema, anasarca, inflammation) stimuli (Campa, Colognesi, et al., 2022; Lukaski et al., 2019; Lukaski & Raymond‐Pope, 2021; Piccoli et al., 1994). However, when decreases in R are accompanied by proportionally greater decreases in Xc, as observed in the current study (R_Whole_: −6.5%; Xc_Whole_: −11.4%), the bioelectrical impedance vector is shifted, resulting in a decrease in PhA (Campa, Colognesi, et al., 2022) (Figure 1). Previous studies report that low PhA is associated with loss of lean body mass, muscle strength, and quality of life, as well as malnutrition, alcoholism, aging, cancer/cachexia, frailty, and mortality, among others (Cardinal et al., 2010; Fukuoka et al., 2022; Genton et al., 2017, 2018; Gupta et al., 2008; Lukaski et al., 2017; Nescolarde et al., 2013; Tanaka et al., 2019; Toso et al., 2000). Within that context, the reductions in R, Xc, Z, and PhA observed in the current investigation likely indicate an inability of cells to store energy, body cell mass loss, inflammation, cellular membrane disruption, and negative energy balance. That these observations persisted through REC for all variables except PhA_Whole_ highlights the longitudinal implications of sustained military operations, demonstrating that cellular integrity is impaired for several weeks into recovery.

Changes in physical performance have been previously reported in the larger cohort of subjects included in this study (Varanoske et al., 2022) but were reanalyzed here to include only subjects in the BIA analysis. Performance on the vertical jump, 3‐RM deadlift, Wingate, and ruck march time trial tests were impaired at POST compared to PRE. Wingate peak power, VO_2peak_, and ruck march time trial time improved from POST to REC. At REC, performance on all tests were not different from PRE. However, the reported changes in physical performance were not different between TEST and PLA. Reasons for the lack of a beneficial effect of TA on performance may be due to the short study duration (Saad et al., 2011; Varanoske et al., 2020), the training status of participants, the high musculoskeletal injury rate during the simulated military operation which may not have permitted subjects to perform to the best of their ability, the potential lack of motivation during performance testing, or several other factors detailed extensively in our previous report (Varanoske et al., 2022). Importantly, despite no effect of TA on physical performance, TEST maintained FFM throughout the simulated military operation, suggesting that TA may have additional beneficial effects on muscle before physical performance improvements are observed (Varanoske et al., 2022).

However, contrary to our hypothesis, reductions in R, Xc, Z, and PhA from PRE to POST were not attenuated in TEST compared to PLA. As research suggests that lean body mass is positively associated with PhA (Francisco et al., 2020; Norman et al., 2012; Primo et al., 2022) and circulating testosterone concentrations are positively associated with PhA, body cell mass, ICW, and FFM in males with chronic kidney disease (Cigarran et al., 2013), it is surprising that the greater FFM and total and free testosterone concentrations in TEST (Varanoske et al., 2022) did not translate to improvements in R, Xc, Z, or PhA. Nevertheless, correlation should not imply causation, and it is possible that factors other than androgen status contribute to changes in PhA. Testosterone exerts its anabolic biological effects by binding to the androgen receptor in the cell cytoplasm, triggering a cascade of events to induce expression of genes specific to promote the growth and development of male sex organs and secondary male sexual characteristics, including changes in muscle and fat distribution as a result of increased muscle protein synthesis and reduced breakdown (Ferraldeschi et al., 2015; Handelsman, 2013; Nieschlag & Nieschlag, 2019; Rossetti et al., 2017) that may act independently of cellular membrane health. While other studies suggest that TA may also act as an anti‐inflammatory agent (Altuwaijri et al., 2003; D'Agostino et al., 1999; Urban et al., 2014) which may decrease proteolysis (Bhatnagar et al., 2012; Dogra et al., 2006; Langen et al., 2004) as well as an anti‐apoptotic agent to prolong cell survival in culture models (Erkkila et al., 1997; Kang et al., 2021; Morimoto et al., 2005; Pronsato et al., 2012), we reported observed no effect of TA on cellular membrane integrity. Potential reasons for this discrepancy may be related to the non‐invasive technique of assessing cellular health in the current study versus using more invasive techniques of obtaining biological samples to examine gene and protein expression, mitochondrial transcription, immunohistochemistry, inflammation, and cell morphology that may provide additional insight. It is possible that BIA parameters are not sensitive enough to discern potential effects of TA on cellular membrane integrity in this cohort. Nevertheless, all participants in the current study underwent the same relative physiological stress during the simulated military operations, and TEST and PLA exhibited similar increases in circulating cortisol and insulin‐like growth factor‐1 (IGF‐1) concentrations and decreases in sex‐hormone binding globulin (SHBG) (Varanoske et al., 2022). Therefore, despite maintenance of muscle mass in TEST, sustained military operations appears to elicit a similar extent of muscle damage and disruption to cellular membrane integrity in both TEST and PLA, and the mechanism of testosterone action likely increases muscle mass independently of improvements in cellular health and integrity.

Significant positive associations were observed between PhA_Whole_ and performance on the vertical jump, 3‐RM deadlift, and Wingate tests, as well as between PhA_Legs_ and vertical jump height at PRE. These observations align with several cross‐sectional studies demonstrating that higher PhA is related to greater strength and power performance and that elite athletes generally possess greater PhA than control counterparts (Cioffi et al., 2020; Custodio Martins et al., 2022; Fukuoka et al., 2022; Giorgi et al., 2018; Langer et al., 2022; Micheli et al., 2014; Norman et al., 2011; Piccoli et al., 2007; Rodriguez‐Rodriguez et al., 2016; Sato et al., 2020). Although a direct cause‐effect relationship has not been established, positive correlations between PhA_Whole_ and Xc_Whole_ and negative correlations between PhA_Whole_ and R_Whole_ are consistent with the notion that strength training interventions elicit increases in FFM and body cell mass and decreases in fat mass, altering the electrical conductivity of tissues (Mulasi et al., 2015). A recent meta‐analysis demonstrated that resistance training elicits a leftward shift in the bioelectrical impedance vector as a result of decreases in R and increases in Xc, whereas physical inactivity induces a rightward shift (Campa, Colognesi, et al., 2022). Tissues with greater water and electrolyte content act as better conductors, decreasing R; simultaneously, increases in FFM stimulate increases in Xc (Mulasi et al., 2015; Ribeiro et al., 2018). Together, increases in Xc and decreases in R increase PhA (Custodio Martins et al., 2022). These inferences are further supported by the significant positive correlations between Xc (in both Whole and Legs) and vertical jump height, and negative correlations between Z and R (in both Whole and Legs) and 3‐RM deadlift mass and Wingate peak power at PRE. These results highlight the contribution of tissue conductive properties to strength and power performance.

Despite these findings, performance on the ruck march time trial task was not significantly associated with any BIA measure, and VO_2peak_ was only significantly associated with Z_Legs_ and R_Legs_, but not with any parameter in Whole. In contrast, other studies report significant associations between PhA and aerobic capacity in adolescents, older adults, and obese individuals (Langer, da Costa, et al., 2020; Langer, de Fatima, et al., 2020; Matias et al., 2020; Streb et al., 2020). As PhA is associated with the number of cells in the body (Dittmar et al., 2015), it is thought that greater cell mass can provide more metabolic activity to generate greater oxygen consumption and carbon dioxide production for physical activity (Fiaccadori et al., 2014; Moore & Boyden, 1963). However, research in healthy, physically‐active adults is lacking, and we are among the first to report relationships between aerobic capacity and PhA in young, athletic, healthy individuals. It is possible that PhA may be predictive of aerobic performance only in populations where cellular health may already be compromised. Additional research examining relationships between PhA and aerobic capacity in young, healthy individuals is warranted.

Although significant reductions in both physical performance and BIA parameters were observed from PRE to POST, no significant correlations were observed between these metrics at POST, ΔPhA was not correlated with changes in any physical performance variable, and only ΔZ_Whole_ and ΔR_Whole_ were associated with Δ3‐RM deadlift mass. These findings contradict longitudinal studies reporting significant relationships between changes in PhA and strength following resistance training interventions (Fukuda et al., 2016; Nunes et al., 2019; Ribeiro et al., 2017; Souza et al., 2017). However, resistance training interventions aimed at improving strength usually incorporate adequate rest, proper nutrition, and progressive overload designed to stimulate muscular adaptations while avoiding overuse and overtraining. In contrast, the current study employed an acute intervention designed to simulate a multi‐stressor military operation, which consisted of high exercise‐induced energy expenditure (up to 12 h of structured physical activity per day), limited energy intake (resulting in an energy deficit), and sleep deprivation (Varanoske et al., 2022). This intervention induced undesirable changes in cellular membrane integrity, consistent with other studies reporting reductions in PhA and Xc following muscle injury (Nescolarde et al., 2013). Despite the persistent suppression of bioelectrical impedance parameters at REC compared to PRE, many of the associations between these parameters and physical performance that were present at PRE once again became significant at REC. Although it is possible that the high incidence of musculoskeletal injury or lack of motivation during the sustained military operation may have affected physical performance testing at POST (Varanoske et al., 2022), these findings suggest that acute impairments in cellular membrane integrity in this cohort are not related to the extent of physical performance decrements. Longitudinal measures of PhA, Xc, R, and Z appear to be more indicative of physical performance rather than acute measures obtained after physiological stress.

We observed greater reductions in BIA parameters measured in the Legs compared to those measured in Whole (R_Whole_: −6.5%, R_Legs_: −10.8%; Xc_Whole_: −11.4%, Xc_Legs_: −18.3%; Z_Whole_: −6.6%, Z_Legs_: −10.9%; PhA_Whole_: −5.0%, PhA_Legs_: −8.6%). These findings may be attributed to the implementation of primarily lower‐body exercises to increase energy expenditure during the simulated military operation. These prolonged, low‐intensity, repetitive aerobic activities, which included ruck marching, running, walking, cycling, and elliptical, likely resulted in greater disruption of cellular integrity in the legs than in the trunk or arms. The inability of PhA_Legs_ to recover to PRE values by REC is also likely a result of a greater disruption in the legs than in the whole‐body during the simulated military operation, and a longer recovery period is likely necessary for complete recovery of PhA_Legs_. However, the associations between raw BIA parameters and physical performance were stronger, and significant relationships occurred more frequently, in Whole than in Legs at PRE and REC, despite the performance tests also relying heavily on the lower body. These findings contradict those in elite soccer players demonstrating that changes in lower‐body PhA from pre‐season through the first half of a championship were a stronger predictor of vertical jump performance than changes in whole‐body PhA (Bongiovanni et al., 2021). However, this study examined longitudinal changes in PhA over the course of a training season where both PhA of the lower‐body and vertical jump height increased, reflecting improved cellular integrity and muscle mass (Bongiovanni et al., 2021). In contrast, the present study examined changes in performance and PhA in response to acute severe physiological stress, reporting decreases in all of these parameters.

Strengths of this investigation included the use of a controlled environment eliciting significant physiological stress, in which energy intake, energy expenditure, and sleep duration were meticulously designed and monitored. Additionally, this study appears to be the first to examine changes in raw BIA vectors in response to military operations as well as TA in young, healthy males, demonstrating that cellular membrane integrity is impaired during sustained military operations, but these effects are not attenuated by TA. The use of raw BIA parameters, rather than those that are calculated from prediction equations (ex: TBW, FFM, ICW, ECW), enhances our confidence in the accuracy of the measures as these are not based on assumptions of cellular hydration. Nevertheless, several limitations should be noted. The observational study design prevents us from drawing conclusions regarding causality, and we are unable to definitively state that longitudinal changes in PhA contribute to changes in physical performance. Additionally, as this was a secondary investigation of a larger study, this analysis may be underpowered to detect relationships between some physical performance metrics and bioelectrical impedance parameters. Also, the number of correlations included in this analysis increase risk for type II error, and this investigation should therefore be considered preliminary and hypothesis‐generating. Furthermore, this study recruited only male participants, so the findings are likely not generalizable to females, and future research examining the effects of sustained military operations on cellular integrity in females is warranted. Finally, the use of a single timepoint for BIA analyses during REC, where most variables did not return to PRE values by the end of this period, does not allow us to provide a time course of recovery. The smallest relative decrement reported in any BIA variable from PRE to POST was PhA_Whole_ (−5.0%), which was also the only variable to return to PRE values by REC, and it is possible that a longer recovery period is necessary for other BIA parameters with larger decrements to return to baseline values.

In conclusion, this analysis demonstrated that sustained simulated, sustained military operations elicit significant disruptions to cellular membrane integrity, as evidenced by decreases in PhA in both the whole‐body and legs. The shift in PhA was a result of decreases in Xc and increases in R, indicating reduced cellular mass, an inability of cells to store energy, and inflammation. TA (administered once, 750 mg testosterone undecanoate) did not attenuate changes in any of these parameters despite the maintenance of FFM, as previously reported (Varanoske et al., 2022). PhA was positively associated with indices of strength and power (vertical jump height, 3‐RM deadlift mass at PRE, and Wingate peak power), but not aerobic capacity (VO_2peak_, ruck march time trial time) at PRE and REC. However, PhA was not associated with physical performance at POST, and changes in PhA from PRE to POST were not associated with changes in physical performance. These findings suggest that sustained military operations elicit cellular membrane disruption, but TA does not mitigate these consequences. Additionally, greater PhA is indicative of greater strength and power performance, but is not related to aerobic capacity in healthy, young males. These relationships also do not exist during periods of acute stress or muscle damage.

## AUTHOR CONTRIBUTIONS

Alyssa N. Varanoske, Neil M. Johannsen, Steven B. Heymsfield, Frank L. Greenway, Jennifer C. Rood, Arny A. Ferrando, and Stefan M. Pasiakos conceived and designed research; Alyssa N. Varanoske, Melissa N. Harris, Callie Hebert, Steven B. Heymsfield, Frank L. Greenway, and Jennifer C. Rood performed experiments; Alyssa N. Varanoske and Steven B. Heymsfield analyzed data; Alyssa N. Varanoske interpreted results of experiments; Alyssa N. Varanoske prepared figures; Alyssa N. Varanoske drafted manuscript; Alyssa N. Varanoske, Neil M. Johannsen, Steven B. Heymsfield, Arny A. Ferrando, and Stefan M. Pasiakos edited and revised manuscript; Alyssa N. Varanoske, Melissa N. Harris, Callie Hebert, Neil M. Johannsen, Steven B. Heymsfield, Frank L. Greenway, Arny A. Ferrando, Jennifer C. Rood, and Stefan M. Pasiakos approved final version of manuscript.

## FUNDING INFORMATION

The U.S. Army Medical Research and Development Command, Military Operational Medicine Research Program funded this research. Supported in part by an appointment to the U.S. Army Research Institute of Environmental Medicine administered by the Oak Ridge Institute for Science and Education through an interagency agreement between the U.S. Department of Energy and the U.S. Army Medical Research and Development Command. The funding sources had no role in the study design; collection, analysis, and interpretation of data; in writing the report; and in the decision to submit this article for publication.

## CONFLICT OF INTEREST STATEMENT

The authors declare that they have no competing interests. The opinions or assertions contained herein are the private views of the authors and are not to be construed as official or as reflecting the views of the Army or the Department of Defense. Any citations of commercial organizations and trade names in this report do not constitute an official Department of the Army endorsement of approval of the products or services of these organizations.

## ETHICS STATEMENT

The Institutional Review Board (IRB) of the PBRC (protocol 2019‐017) and the U.S. Army Medical Research and Development Command, Human Research Protections Office approved the study protocol and trial documents, and all procedures were in accordance with the ethical standards of the 1964 Helsinki Declaration and its later amendments.
